# Inappropriate Administration of Rabies Postexposure Prophylaxis, Cook County, Illinois, USA

**DOI:** 10.3201/eid2610.200232

**Published:** 2020-10

**Authors:** Hannah D. Steinberg, Kelley Bemis, Mabel M. Frias, Demian Christiansen

**Affiliations:** Cook County Department of Public Health, Forest Park, Illinois, USA (H.D. Steinberg, K. Bemis, M.M. Frias, D. Christiansen);; Council of State and Territorial Epidemiologists, Atlanta, Georgia, USA (H.D. Steinberg)

**Keywords:** rabies, postexposure prophylaxis, quality improvement, viruses, zoonoses, Illinois, United States

## Abstract

Administration of rabies postexposure prophylaxis (PEP) is expensive and time-consuming. In suburban Cook County, Illinois, USA, administration of 55.5% of PEP treatments did not follow Advisory Committee on Immunization Practices guidelines. Health department consultation lowered the odds of inappropriate PEP administration by 87%. Providers should consult their health department before prescribing PEP.

Rabies is typically fatal to unvaccinated patients; however, the prompt administration of postexposure prophylaxis (PEP) can prevent disease onset (*1*). When a patient is exposed to a potentially rabid animal, that patient’s physician must determine whether administration of PEP is prudent. The Advisory Committee on Immunization Practices (ACIP) publishes guidelines indicating when physicians should administer PEP (*1**,**2*). Lack of adherence to these guidelines might result in unnecessary costs and medical risks (e.g., injection site reactions, systemic hypersensitivity reactions) (*1**,**3*). In the United States, a full course of PEP (usually 4 vaccine doses and 1 immunoglobulin dose [*2*]) costs $3,800 on average (*4*). In Illinois, the patient, their insurance provider, or both pay for PEP. Illinois physicians must report PEP initiation to local public health departments (*5*). 

We retrospectively evaluated patients who received PEP in suburban Cook County, Illinois, during 2015–2018 and were reported to the Cook County Department of Public Health (CCDPH). Although Chicago is in Cook County, it has its own health department and was therefore not included in this study. We used a multivariable logit link generalized estimating equation model (*6*) to evaluate predictors of inappropriate PEP administration according to ACIP guidelines. We analyzed factors such as patient age, patient sex, area of residence, exposing animal species, and whether a state or local health department was consulted before PEP initiation. We controlled for clustering by exposure incident (i.e., multiple persons exposed to the same animal) by using robust variance estimators and assuming an independent correlation structure. We conducted statistical analyses in R version 3.5.3 (*7*) and ran models using geepack version 1.2–1 (*6*). Because the purpose of this study was to evaluate and inform public health practices, it was not considered human subjects research by the Cook County Health Office of Research and Regulatory Affairs and was exempt from institutional board review.

During 2015–2018, a total of 656 residents initiated PEP. We excluded 45 cases because of missing data; these cases were proportionally distributed in time and geographic area. Of the 611 patients, 339 (55.5%) did not meet ACIP guidelines for potential rabies exposures (Table), a proportion that aligns with previously reported ranges in other US jurisdictions (*8*). The 5 most common reasons for inappropriate PEP administration: 1) the patient had a bat in their home but no known contact with the bat and the patient did not wake to the bat in their room (187 persons); 2) PEP was given after a provoked bite from a dog or cat with no signs of rabies (85 persons); 3) the animal involved was available for confinement or testing (18 persons); 4) the patient had no known animal contact (17 persons); and 5) the animal involved tested negative for rabies (16 persons).

**Table:** PEP recipients and factors associated with inappropriate administration of PEP, suburban Cook County, IL, 2015–2018*

Variable	Total, no. (%), n = 611	Exposure met ACIP guidelines for PEP administration, no. (%)		Unadjusted GEE model† OR (95% CI)	Adjusted GEE model‡ aOR (95% CI)
		Yes, n = 272	No, n = 339		
District§					
North	309 (50.6)	125 (45.9)	184 (54.3)	Referent	Referent
West	131 (21.4)	54 (19.9)	77 (22.7)	0.97 (0.52–1.80)	0.76 (0.39–1.47)
Southwest	82 (13.4)	47 (17.3)	35 (10.3)	0.51 (0.27–0.94)	0.41 (0.20–0.83)
South	89 (14.6)	46 (16.9)	43 (12.7)	0.64 (0.35–1.15)	0.52 (0.27–0.98)
Age, y					
0–5	47 (7.7)	24 (8.8)	23 (6.8)	0.84 (0.44–1.62)	0.74 (0.36–1.50)
6–17	170 (27.8)	64 (23.5)	106 (31.3)	1.46 (0.92–2.32)	1.49 (0.90–2.45)
18–25	50 (8.2)	23 (8.5)	27 (8.0)	1.03 (0.55–1.94)	1.20 (0.53–2.72)
>26	344 (56.3)	161 (59.2)	183 (54.0)	Referent	Referent
Sex					
F	317 (51.9)	131 (48.2)	186 (54.9)	Referent	Referent
M	294 (48.1)	141 (51.8)	153 (45.1)	0.76 (0.53–1.10)	0.77 (0.51–1.15)
Exposing animal					
Bat	393 (64.3)	181 (66.5)	212 (62.5)	Referent	Referent
Cat	35 (5.7)	6 (2.2)	29 (8.6)	4.13 (1.62–10.50)	4.15 (1.49–11.60)
Dog	111 (18.2)	39 (14.3)	72 (21.2)	1.58 (0.91–2.72)	2.05 (1.07–3.96)
Raccoon	31 (5.1)	26 (9.6)	5 (1.5)	0.16 (0.06–0.45)	0.19 (0.06–0.57)
Other	41 (6.7)	20 (7.4)	21 (6.2)	0.90 (0.45–1.79)	0.93 (0.43–2.01)
HD consult¶					
Yes	183 (30.0)	138 (50.7)	45 (13.3)	0.15 (0.09–0.23)	0.13 (0.08–0.22)
No	428 (70.0)	134 (49.3)	294 (86.7)	Referent	Referent

*ACIP, Advisory Committee on Immunization Practices; aOR, adjusted odds ratio; GEE, generalized estimating equation; HD, health department; PEP, rabies postexposure prophylaxis; OR, odds ratio. †Bivariate GEE model for PEP inappropriateness as a function of the given categorical variable. ‡Multivariable GEE model for PEP inappropriateness as a function of all the predictors included in the table. §Suburban Cook County residential district of patient’s home address. ¶Whether healthcare provider contacted a state or local health department to discuss appropriateness of PEP.

The results of the generalized estimating equation model showed that provider consultation with the health department, species of the exposing animal, and patient area of residence were factors associated with appropriate administration of PEP (Table). The most protective factor against inappropriate PEP administration was a health department consultation, a service CCDPH offers free of charge 24 hours a day, 7 days a week. After adjusting for patient age, sex, area of residence, and exposing animal, we found patients who received PEP were 87% less likely to have received inappropriate treatment if their healthcare provider consulted a health department (adjusted odds ratio [aOR] 0.13, 95% CI 0.08–0.22). Because 428 patients (70.0%) received PEP without health department consultation, this service could be used to reduce the unnecessary administration of PEP.

Certain animal species were also associated with inappropriate PEP administration. We found greater odds of inappropriate PEP administration associated with exposure to dogs (aOR 2.05, 95% CI 1.07–3.96) and cats (aOR 4.15, 95% CI 1.49–11.60) than bats. Exposure to raccoons was associated with reduced odds of inappropriate PEP administration (aOR 0.19, 95% CI 0.06–0.57) (Table). The reason for this pattern might be that ACIP guidelines are more complicated for domestic than wild animal exposures (e.g., determining whether a bite was provoked). Health departments can assist providers with these determinations before initiating PEP.

Patient residential district was associated with inappropriate PEP administration, whereas patient age and sex were not (Table). This finding suggests additional local factors might exist, such as differences in wealth, cost-aversion, or rabies awareness, for which we did not control in our estimates.

PEP is an expensive and time-consuming treatment. Although clinicians should encourage PEP for patients with potential exposures to rabies, they should avoid it when risk for rabies does not exist (*1*). Health departments around the United States follow the ACIP guidelines for recommending PEP (*1**,**2*) and have unique knowledge of their local rabies epidemiology. Providers should consider the benefits and risks of PEP and consult their health department before prescribing PEP.

## References

[1] Manning SE, Rupprecht CE, Fishbein D, Hanlon CA, Lumlertdacha B, Guerra M, et al.; Advisory Committee on Immunization Practices Centers for Disease Control and Prevention. Human rabies prevention—United States, 2008: recommendations of the Advisory Committee on Immunization Practices. MMWR Recomm Rep. 2008;57:1–28. 18496505

[2] Rupprecht CE, Briggs D, Brown CM, Franka R, Katz SL, Kerr HD, et al.; Centers for Disease Control and Prevention. Use of a reduced (4-dose) vaccine schedule for postexposure prophylaxis to prevent human rabies: recommendations of the Advisory Committee on Immunization Practices. MMWR Recomm Rep. 2010;59:1–9. 20300058

[3] Christian KA, Blanton JD, Auslander M, Rupprecht CE. Epidemiology of rabies post-exposure prophylaxis—United States of America, 2006-2008. Vaccine. 2009;27:7156–61. 10.1016/j.vaccine.2009.09.028 19925946

[4] Centers for Disease Control and Prevention. Cost of rabies prevention. 2019 [cited 2019 Jul 16]. https://www.cdc.gov/rabies/location/usa/cost.html

[5] Illinois General Assembly-Joint Committee on Administrative Rules. Illinois administrative code, title 77, part 690, section 690.601: rabies, potential human exposure and animal rabies. 2014 [cited 2019 Jul 16]. http://www.ilga.gov/commission/jcar/admincode/077/077006900D06010R.html

[6] Halekoh U, Højsgaard S, Yan J. The R package geepack for generalized estimating equations. J Stat Softw. 2006;15. 10.18637/jss.v015.i02

[7] R Core Team. R: a language and environment for statistical computing. Vienna (Austria): R Foundation for Statistical Computing; 2019.

[8] Moran GJ, Talan DA, Mower W, Newdow M, Ong S, Nakase JY, et al.; Emergency ID Net Study Group. Appropriateness of rabies postexposure prophylaxis treatment for animal exposures. JAMA. 2000;284:1001–7. 10.1001/jama.284.8.1001 10944646

